# Exploring interprofessional, interagency multimorbidity care: case study based observational research

**DOI:** 10.15256/joc.2017.7.103

**Published:** 2017-06-12

**Authors:** Eileen M. McKinlay, Sonya J. Morgan, Ben V. Gray, Lindsay M. Macdonald, Susan R.H. Pullon

**Affiliations:** ^1^Department of Primary Health Care and General Practice, University of Otago, Wellington, New Zealand

**Keywords:** Communication, interagency, interprofessional interactions, multimorbidity, collaboration, consultation, coordination

## Abstract

**Background:**

The increase in multimorbidity or co-occurring chronic illnesses is a leading healthcare concern. Patients with multimorbidity require ongoing care from many different professionals and agencies, and often report a lack of integrated care.

**Objective:**

To explore the daily help-seeking behaviours of patients with multimorbidity, including which health professionals they seek help from, how professionals work together, and perceptions and characteristics of effective interprofessional, interagency multimorbidity care.

**Design:**

Using a case study observational research design, multiple data sources were assembled for four patients with multimorbidity, identified by two general practitioners in New Zealand. In this paper, two case studies are presented, including the recorded instances of contact and communication between patients and professionals, and between professionals. Professional interactions were categorized as consultation, coordination, or collaboration.

**Results:**

The two case studies illustrated two female patients with likely similar educational levels, but with different profiles of multimorbidity, social circumstances, and personal capabilities, involving various professionals and agencies. Engagement between professionals showed varying levels of interaction and a lack of clarity about leadership or care coordination. The majority of interactions were one-to-one consultations and rarely involved coordination and collaboration. Patients were rarely included in communications between professionals.

**Conclusion:**

Cases constructed from multiple data sources illustrate the complexity of day-to-day, interprofessional, interagency multimorbidity care. While consultation is the most frequent mode of professional interaction, targeted coordinated and collaborative interactions (including the patient) are highly effective activities. Greater attention should be given to developing and facilitating these interactions and determining who should lead them.

## Introduction

Chronic co-occurring illnesses are a leading healthcare concern worldwide, including in New Zealand [1, 2]. Patients with multimorbidity are defined as those “*having any combination of chronic disease with at least one other disease (acute or chronic) or biopsychosocial factor (associated or not) or somatic risk factor*” [3]. The acknowledgement that biosocial and environmental factors have a causative and/or additive impact on the clinical diseases associated with multimorbidity recognizes that these factors interact synergistically, particularly in relation to those who are vulnerable or live in poverty [4]. These patients are recognized as requiring frequent, regular support for ongoing needs from a range of health and social care professionals usually associated with different agencies [5], often over many years [6]. These patients also seek support from those considered as non-traditional professionals [7]. Some needs are straightforward and require the province of one profession, whereas others require more complex and often simultaneous interactions involving the patient, professionals (generalists and specialists) and different agencies, including those in primary, secondary, and tertiary health settings, as well as in social care settings. Leadership is often unclear when different professionals, disciplines, and agencies are involved [8, 9]. It has been suggested that general practice (and particularly general practitioners [GPs]) are well placed to be the lynchpin to provide leadership and continuity of patient-centred care, to involve the patient in the care team, and to foster self-management skills [10–12].

However, there are known barriers when implementing interprofessional, interagency approaches, and patients with multimorbidity report ‘gaps’, fragmentation, duplication, and disparate forms of health and social care [13–16]. In particular, they note the time-consuming and costly aspects of coordinating care, and difficulty in reconciling differing professional advice and interventions (particularly medications). Patients also comment on the difficulty of judging the severity of each illness or recognizing which illness is currently causing the most significant health problems, and whether or which self-management strategies can help single or multiple illnesses [17]. All of these concerns determine if, or when, they seek professional assistance, and if so, which profession, generalist, specialist, or agency is best placed to help them [13, 18–21].

There is limited theory to guide the development of models of care for people with multimorbidity involving professionals working across agencies [22–24]. The ‘3Cs’ non-hierarchal model of clinician interpersonal interactions, including *consultation, coordination,* and* collaboration*, appears to have conceptual relevance [22]. The model was developed from a comprehensive study of 19 diverse American-based primary care practices involving 160 intensive direct observations of actual interactions between primary care and behavioural healthcare clinicians during patient visits (followed by 90 interviews), with the aim of describing the delivery of integrated care across 19 diverse integrated settings [22]. 

There has also been little, if any, previous research using case study based observational methods undertaken in real-world settings to explore interprofessional, interagency multimorbidity care. Observational methods in which data are contemporaneously collected in real-life settings, although challenging to conduct, have the potential to provide robust information about interprofessional, interagency multimorbidity care. They reach beyond retrospective self-report studies and reveal the real-world practices of patients, professionals, and agencies [25]. To address this research gap, the present study aimed to examine the following:

1. The daily help-seeking behaviours of patients with multimorbidity, including the answers to the questions: Who do they contact? How do they do it?
2. The frequency and means by which general practice based professionals, such as GPs, interact with professionals in other health agencies when managing such patients, and whether patients are aware of these interactions.
3. What patients and professionals consider to be effective interprofessional, interagency multimorbidity care.

### Context and setting

Specific studies on the prevalence of multimorbidity in New Zealand have not been undertaken. However, New Zealand is likely to have comparable numbers of those with multimorbidity to similar countries, such as Australia (one-third of the population), as the rates of long-term conditions are similar [26, 27]. In New Zealand, multimorbidity is likely to have a greater impact on Māori (New Zealand’s indigenous people), who have higher rates of chronic illness and overall lower life expectancy [28].

New Zealand provides no-charge secondary and tertiary medical specialist and hospital-level care, but there is a part-charge for patients to obtain first-contact general practice services (GPs and practice nurses) through a mixed capitation/fee-for-service funding model. Professionals, such as counsellors, medical specialists, midwives, physiotherapists, and social workers, work in either private or public health and social care agencies, with private agencies charging for service [29].

In the 1990s, the New Zealand government introduced lead maternity carers (LMCs) to provide funded maternity services. LMCs are either midwives, obstetricians, or GP obstetricians (there are now very few of the latter), and are chosen by the pregnant women to lead their maternity care. Some women attend GPs for confirmation of pregnancy and early antenatal care, but most approach an LMC from the start of their pregnancy. It has been argued that this form of maternity care isolates the LMC and disrupts the usual continuity of care provided by general practices [30].

New Zealand general practice and public hospital staff use different electronic health record (EHR) platforms. These platforms are used to record consultations, other interactions with and about patients, and are a repository for scanned letters, laboratory, radiological, and other test results. Some general practice and public hospital staff have limited access to each other’s patient EHRs. Electronic patient portals (patient e-portals; providing secure online access to personal health information) have recently been introduced in general practice settings [29].

## Methods

A multiple case study design [31] using case study observational research (CSOR) methods [25] was used to explore the research questions within real-world settings. CSOR specifies a sequential order for data collection, data analysis, and synthesis, starting with (direct or indirect) observational methods. It is also an inductive iterative process, with the analysis of observational data informing the collection of other self-report data (e.g. surveys, interviews, or documentation review).

In the current study, within the 12–13-week study period, data collection methods included interviews (see Supplementary Methods), health encounter diaries (see Supplementary Methods for template), portions of EHRs, and other written documents. Four patient cases were developed and two were then analysed in detail to generate conclusions. The study was undertaken in 2014 and received ethical approval from the University of Otago Health Ethics Committee (approval no. H14/002).

### Participants

Participants included two GPs, four patients, and three nominated professionals from different agencies; nine participants in total. The two GPs (GP1 and GP2), both experienced part-time female GPs (a typical profile for a New Zealand GP) [32] from two diverse practices (in terms of location, patient demographics, and business models), were approached, and both agreed to participate. 

The two GPs were each asked to identify two patients with multimorbidity. The definition of multimorbidity, as defined by Le Reste *et al*. [3], informed the inclusion criteria of the study:* “at least two ongoing chronic conditions requiring frequent care from their GP and at least one other health or social service agency, and be able to keep a diary for 12–13 weeks”.* Each GP requested agreement from the patients to be contacted by a researcher. A research nurse then contacted the patients to explain the study and obtain their consent.

The GPs used purposeful sampling of patients to identify information-rich cases [33]. All four patients agreed to participate and complete a diary of health contacts over 12–13 weeks; no patient declined to participate. The patients were asked to nominate a professional from another agency with whom they had frequent contact during the diary collection period to be part of the study. All of those nominated were healthcare professionals and all agreed to participate. One patient did not have sufficient contact with professionals in other agencies during the data-collection period to nominate another participant. Informed consent was obtained from all participants.

### Procedures

**Figure 1:**
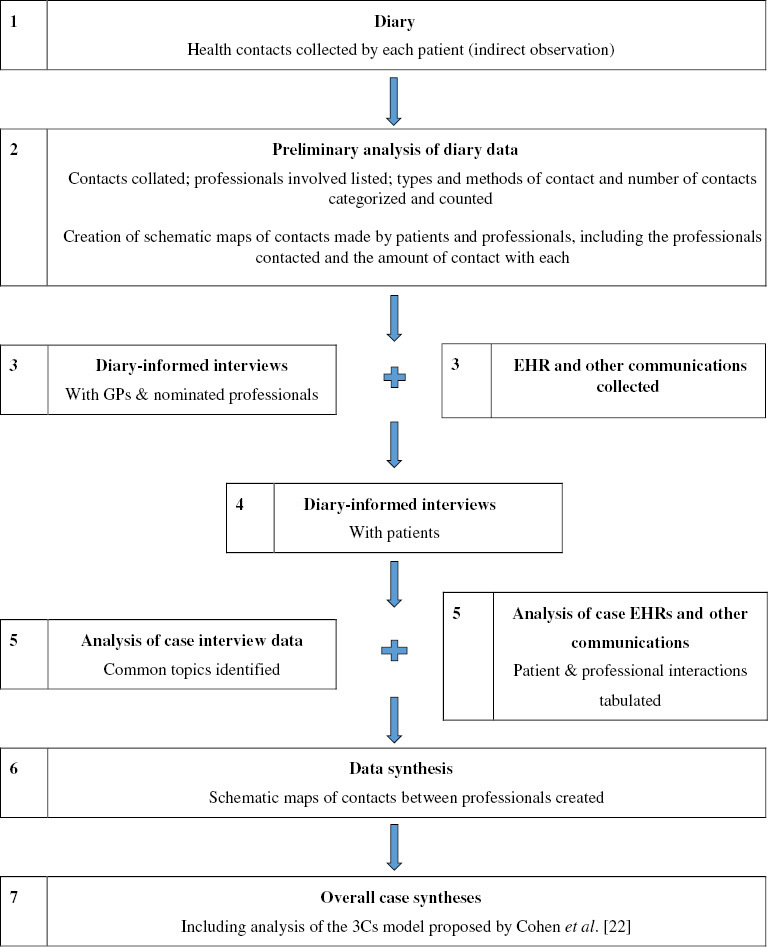
Sequence of data collection, analysis, and synthesis. EHR, electronic health record; GPs, general practitioners.

Data for each patient were collected from four key sources, as outlined below (see Figure 1 for the sequence of data collection, analysis and synthesis).

***1. Patient health encounter diary (indirect observation)***

The patients completed a 12–13-week structured health encounter diary [34], which was chosen as an effective indirect observational method [35], as direct observation was thought to be unfeasible and unacceptable to patients over a prolonged period of time [36]. Methods of indirect observation, such as diaries and similar daily records, are considered comparable to those of direct observation [37] (the visual observation of individual participants in natural settings [38]).

The diary developed by the research team was piloted with two test patients and refined. It comprised a template of two distinct sections. Part 1 was a daily assessment of general health recorded on a 5-point scale, and a question about whether or not there had been any health contact made. Part 2 was an opportunity for guided free text entries of any health contact that occurred either face-to-face, by phone, text, letter, patient e-portal or email. Patients were also asked to comment on whether or not they thought the health or social carers might have communicated with each other. The ethnographically trained research nurse, who had extensive experience in collecting interview and observational data in primary care settings, kept in regular contact with the patients during the diary-keeping period, visiting in the initial weeks and then following up with phone calls.

***2. Diary-informed interviews***

Following analysis of the patients’ health encounter diaries, the research team developed an unstructured interview guide [39] to conduct individual face-to-face audio-recorded interviews with the patients and GPs. In contrast to semi-structured interviews, where interviews are often the sole data source and are based on a set of largely fixed questions, the unstructured interviews with the research nurse took the form of ‘guided conversations’ [31, 39]. These conversations centred on the completed diary, which was reviewed during the conversation. By the time the diary was completed, the research nurse was well known to each patient and had maintained field notes of multiple conversations during the diary-collection period. Drawing on information from her previous conversations, she was readily able to query the content of the diary with the patients and, similarly, the EHRs with the GPs. This resulted in the conversation guide prompts sometimes referring to previous information or building on an opinion already voiced. 

The patient conversation guide included prompts to the patient about their role in managing their own health; the role of the professionals in their care; perceptions of communication, coordination, and collaboration; and the experience and acceptability of diary keeping. The GP and other professional conversation guides included prompts to the GP or other professional about their role in communication, coordination, and collaboration between professionals; views of barriers and facilitators to effective collaboration; and the experience and acceptability of the methods. 

***3. Selected portions of the EHR***

At completion of the interviews with professionals, EHR database fields containing records of each patient’s care were extracted for the dates of the diary-keeping period. These records included clinical consultation notes, related follow-up records, medications, letters/emails of follow-up from correspondence or referral between general practice (GPs and practice nurses) and other professionals, and all laboratory and radiological results. Personal identifying information was removed from the copies collected.

***4. Other forms of written communication***

Written communications from the nominated professionals to other professionals not held in the general practice EHR were collected at completion of the interviews. These included email and letter communications. Personal identifying information was removed from all communications.

### Sequence of data collection, analysis, and synthesis

In accordance with CSOR methodology, the data collected through indirect observation were analysed prior to the collection and analysis of non-observational data (Figure 1). Completed patient diaries were entered into a database. Interviews were transcribed and then analysed by two researchers using inductive, iterative thematic analysis, with the first researcher coding according to topic area, and the second researcher analysing selected topics (e.g. working in partnership, collaboration) for themes that were then discussed and agreed upon by the research team [40]. The selected portions of the EHR and other forms of written communication were entered into databases (the interprofessional interaction data). To ensure rigor of the CSOR methodology of undertaking separate and sequential analyses of multiple methods, all data sources, databases, and analyses remained separate before purposefully integrating the results [41]. 

The interprofessional research team categorized the interactions according to the 3Cs model proposed by Cohen *et al*. (developed to explain the nature of integrated care and therefore likely to be applicable to, and explicate, the possible elements of interprofessional, interagency, collaboration) [22]. The categories used were as follows: *consultation* – being advice-seeking and advice-giving; *coordination* – being separate, but aligned, care delivery; and *collaboration* – being shared sense-making and decision-making [22]. These categories were then further tested by corroborating with the interview data. 

## Results

All four patients completed their health encounter diaries over the 12–13-week data collection period (the number of days recorded ranged from 84 to 93, depending on when each diary commenced) (Table 1). Although patients were instructed to notate a written entry each day, even if no professional had been contacted, patients did not always indicate when they had nothing to report. When a contact was reported, some patients provided more comprehensive and detailed information than others.

### Selected cases

For the current study, data of two patients who best illustrated the complexity of multimorbidity are presented (Patients 1 and Patient 2). E.M.M. and S.J.M. selected the two patients for analysis, as they met the criteria for seeing other professionals and also agencies in addition to their GP in the 12–13-week diary-collection period. The selection was reviewed and verified by all authors. The remaining two patients, either did not see any other professional/agency or saw only one professional/agency in the 12–13-week diary-collection period. Box 1 shows brief case overviews of Patient 1 and Patient 2, and describes the general practices involved; this summarizes all of the information known about the patients at the project start. The characteristics of the two patients reflect some of the diversity of those with multimorbidity, including age, number and type of conditions, as well as social, economic, and environmental complexity. 

### Daily contacts and communication between the two patients and professionals

Using the health encounter diaries, we identified the professionals/agencies with whom the patients made contact over the data collection period and the number of contacts made with each professional (see Table 2).

Patient 1 used five different methods to contact professionals/agencies who she herself classified as either “*health*” or “*wellness*” professionals, the latter usually complementary alternative medicine (CAM) professionals. She recorded 55 contacts with 18 professionals/agencies, with varying numbers of contacts with each.

Patient 2 used two different methods of contact and recorded 17 contacts with six different professionals/agencies with varying numbers of contacts with each. 

**Figure 2:**
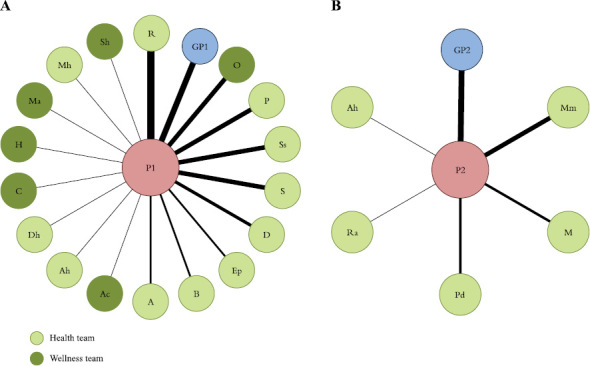
Patient contacts with professionals/agencies recorded during the 12–13-week study period, based on data obtained from the health encounter diaries. A: Patient 1 (P1) contacts with health and wellness professionals; B: Patient 2 (P2) contact with health professionals. Note: denser lines in the figure represent greater number of contacts. A, accident and emergency; Ac, acupuncturist; Ah, after-hours clinic; B, breast clinic; C, counsellor; Dh, dental hygienist; D, dentist; Ep, exercise physiologist; GP1, general practitioner 1, GP2, general practitioner 2; H, homeopath; Ma, masseuse; Mm, maternal mental health; Mh, Ministry of Health; M, midwife; O, osteopath; P, pharmacist; Pd, postnatal depression support^*^; R, rehabilitation clinic; Ra, radiologist; S, sleep clinic; Sh, spiritual healer; Ss, sleep specialist. ^*^Whilst not considered a health provider, the postnatal depression support group was included, as people with multimorbidity are likely to perceive it as having an equal status, especially if using a ‘trained’ facilitator.

Schematic maps were created indicating the extent of the contacts with professionals/agencies, as shown in Figure 2.

As well as recording day-to-day individual contacts with professionals/agencies, patients recorded whether or not professionals/agencies mentioned being in contact with anyone else in relation to their care. Patient 1 reported that she was informed that professionals/agencies had been, or planned to be, in touch in 15 of the 55 contacts. Patient 2 reported that she was informed that professionals/agencies had been, or planned to be, in touch in eight of the 17 contacts. There were other occasions where both patients were uncertain if contact was planned or had even occurred.

In the subsequent interviews with the two patients, the interviewer explored how the patients interacted with the professionals/agencies and if they thought that the professionals/agencies communicated with other professionals/agencies. Patient 1 responded to the interviewer’s question, which referred to a previous conversation about *“holding all the threads”*, and described her role in actively controlling or filtering communication with, and between, professionals involved in her care, depending on the professional’s perceived acceptance of her self-management strategies. Interviewer:* “So do you see yourself as, as holding all the threads? … navigate yourself through all this thing?”* Patient 1:* “Yes. Yes I do. Yes. … I mean I have a superb GP. But I mean I don’t dare mention X* [a wellness professional’s name]* in her presence, you know. … and I didn’t tell* [the GP]* this, or the breast surgeon that … I was having vitamin C infusions when, I was having the* [oncology] *treatment*”.

Patient 2 discussed not knowing if her GP (GP2) communicated with the midwife (her nominated professional) and then responded with a somewhat uncertain response (“*possibly*”) to the interviewer’s query about the possible value of this. Patient 2:* “I don’t think they do* [talk to each other]”*.* Interviewer:* “Would it be good if they did?”* Patient 2:* “I guess possibly at stages there would have been times when it could have been useful. But I don’t know what the outcome would have been… except that that my GP and my midwife would know what’s happening”.*

### Contacts and communications between GPs and other professionals when caring for Patient 1 and Patient 2

An analysis was undertaken of both the contacts and communications recorded in each of the patients’ general practice EHRs, letters, and other correspondence held by the professionals. Based on the total number of professionals involved in the care of each patient (see Tables 2 and 3), there were six recorded contacts between nine of the 24 professionals/agencies for Patient 1, and 22 recorded contacts between 13 of the 14 professionals/agencies for Patient 2. 

The most frequent methods of contact between GPs and other professionals/agencies about the patients were via letter (12 times) and electronic methods (11 times via email or secure electronic messaging). There was one internal face-to-face meeting.

In the interviews with the two GPs, the interviewer explored the GPs’ interaction with other professionals or agencies. GP1:* “*[It’s]* by letter* [to the specialist]*, mostly it’s asynchronous, by written correspondence. That’s the most common, with the patient in the middle, sometimes conveying information, it’s quite uncommon for a GP to be actually speaking to a specialist”.* Interviewer:* “Not a face-to-face?”.* GP1:* “In fact, some of these specialists, I’ve dealt with them for years, and I wouldn’t have met them”.*

In contrast, the nominated professional (physician) working with Patient 1 described attempting to actively communicate with both the patient and the GP, explaining his belief why this was important. Interviewer:* “So do you have a particular view on collaboration, then?* [Patient 1]* thought it was unusual that you included her in the* [GP’s]* letters, and she appreciated that”.* Physician:* “… increasingly I think that’s a good way of communicating… it’s pretty straightforward to actually include the patient in the communication, but it hasn’t been the tradition in medicine”*.

GP2 expressed a general concern about the type and level of interaction that occurred with Patient 2 and the ways in which to improve interactions. Interviewer:* “Has being part of this tiny study… made you think any differently about collaboration?”* GP2: *“Yes it has actually. Because… I don’t think we document this stuff well, I don’t think it would be clear from the outside that this collaboration was happening. So* [it]* definitely made me think, if this is your model, then you need to …document when going to work closely with* [others]*. And we’re going to try and make sure we’ve got those bits of information, being clear that we are collaborating. I’d like to retain some sense of being that coordinating role”.* Interviewer: *“Mm”.* GP2:* “*[currently]* you are just firing out a referral, and getting that expertise to come back… hoping that it will come back to you, so that you can reformulate and move on. … So perhaps being more clear about that, even in the written letter, ‘I want to be kept closely informed about the progress’”.*

Although GP2 desired a more collaborative model of interaction, from the nominated professional’s perspective (midwife), in reality there were barriers to this happening. GP2 acknowledged that she had not made contact with the midwife. GP2:* “I think I remember talking with* [Patient 2]* ‘Do you know who you’re going to go to* [which midwife]*?’. I haven’t had any contact with the midwife… at all, actually”.* Interviewer:* “And they haven’t sought you out?”.* GP2:* “No, they haven’t sought me out or fed back to say, ‘It’s all going well’. When I left it to* [Patient 2]* to contact the midwife, how much information did* [Patient 2]* tell them about where she’s at with her mental health? So I possibly could have closed that loop by checking in with the midwife”.*

### The nature of interprofessional, interagency multimorbidity care

**Figure 3:**
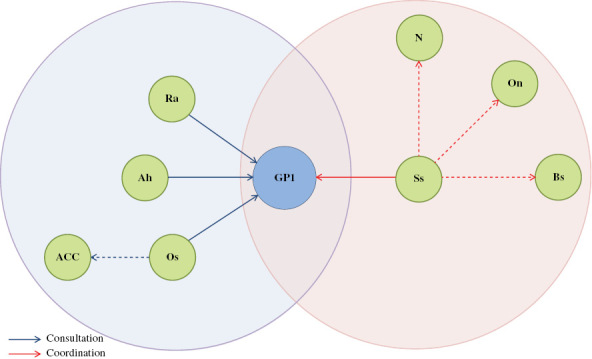
Schematic map of the interaction between the professionals/agencies involved in the care of Patient 1, based on data obtained from electronic health records and written communications from the nominated professionals and interviews. Dashed lines represent communication that was copied to another person. ACC, Accident Compensation Corporation; Ah, after-hours clinic; Bs, breast surgeon; GP1, general practitioner 1; N, neurologist; On, oncologist; Os, orthopaedic surgeon; Ra, radiologist; Ss, sleep specialist.

Information regarding the nature of interprofessional, interagency multimorbidity care was obtained from EHRs and other forms of written communication from the nominated professionals, and interviews. Analysis of all forms of interprofessional communication data resulted in the formation of spheres of interaction revealing the nature of the interprofessional, interagency care. For Patient 1, there were two connected spheres of interaction (see Figure 3). The first sphere included interaction between some of the professionals/agencies in the care of Patient 1 and GP1. Analysis of the documentation showed a one-way communication of information between each professional/agency and the GP, or the use of ‘report-back’ phrases usually in response to a formal referral letter/email, such as *“have filled in the form for* [insurance]*”.* This form of interaction meets the definition proposed by Cohen *et al*. for *consultation* (see arrows marked in blue in Figure 3) [22]. 

The second sphere was between the nominated professional (physician) and other professionals/agencies. Analysis of the interaction showed that, although still mainly unidirectional (in response to contact or referral), it was either responding to and/or inviting further contact. However, in contrast to the consultation form of communication, the written communication used phrases inviting other professionals to align with care, such as,* “She will need a repeat of her* [name of medicine]* which I would be grateful if you could organize”.* This form of interaction meets the definition of *coordination*, as proposed by Cohen *et al*. (see arrows marked in red in Figure 3) [22]. 

**Figure 4:**
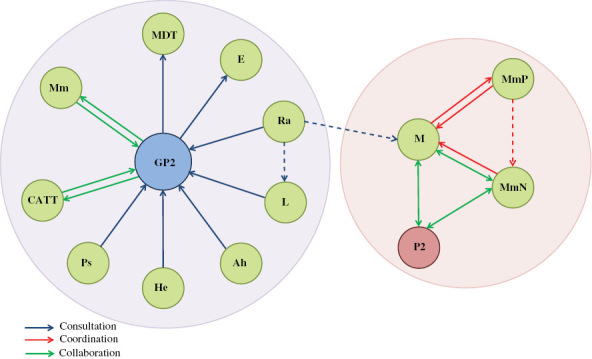
Schematic map of the interactions between the professionals/agencies involved in the care of Patient 2, based on data obtained from electronic health records and written communications from the nominated professionals and interviews. Dashed lines represent communication that was copied to another person. Ah, after-hours clinic; E, employer; GP2, general practitioner 2; He, Healthline (New Zealand’s national telephone health information service); L, laboratory; M, midwife; MDT, multidisciplinary meeting; Mm, maternal mental health; MmN, maternal mental health nurse; MmP, maternal mental health psychiatrist; P2, Patient 2; Ps, psychologist; Ra, radiologist.

For Patient 2, two spheres of interaction were identified, but they were only connected by one e-carbon-copied (cc’ed) communication between two of the professionals (see Figure 4). The first sphere involved GP2 and, similar to Patient 1, included multiple, mainly unidirectional, communications between GP2 and a professional/agency, or between a professional/agency and GP2. Analysis of the interaction showed that, except for two instances (see below), these were one-way (in response to contact or referral), indicating *consultation* (see arrows marked in blue in Figure 4) [22]. However, in contrast to the one-way form of interaction, there were also two interactions in which brief bidirectional exchanges of information occurred. One interaction was a phone call between the practice nurse working with GP2 and the crisis mental health agency (CATT), and was recorded as a file-note in the EHR as, *“Phone call to* [CATT]* to make sure they received the referral and that they are taking care of it”*. A letter from CATT to the practice nurse on that same day reported the actions of the CATT in responding to the referral. The second interaction was between GP2 and the maternal mental health (MMH) agency, and was recorded as a file-note in the EHR as,* “Phone call to maternal mental health to inform* [a clinical event]*”.* Seven days later, a letter from the MMH was sent to the patient and copied to the GP, acknowledging the phone call and noting the agency had confirmed an appointment time with the patient. These interactions (recorded summaries in the EHR and copies of letters) detail joint decisions and actions to be undertaken by the various parties, and meet the definition of *collaboration*, as proposed by Cohen *et al*. (see arrows marked in green in Figure 4) [22]. 

The second sphere of interaction included bidirectional communications between the nominated professional (midwife) and another agency (MMH), including the involvement of two MMH clinicians (psychiatrist, mental health nurse). Analysis of the written documentation (email) showed a reciprocal exchange of information about the care of Patient 2, with one clinician inviting a follow-up contact, *“If you need to get hold of me at any stage you can either call the clinic on* [phone number of agency]* or my cell* [phone number of mental health nurse]*”*. This interaction meets the definition of *coordination*, as proposed by Cohen *et al*. (see arrows marked in red in Figure 4) [22]. However, when this information was augmented by the interview data, it was apparent there had been further unrecorded phone communication between the nominated professional (midwife) and the mental health nurse from the MMH agency in which they had planned a joint meeting together with Patient 2. This interaction meets the definition of *collaboration*, as proposed by Cohen *et al*. (see arrows marked in green in Figure 4) [22].

Despite Patient 1 and Patient 2 experiencing complex multimorbidity, where shared decision-making through coordination or collaboration might have been expected, the majority of the individual interactions between the professionals were by nature, *consultation* alone. There were a few instances in which the interactions with Patient 2 showed *separate, but aligned care delivery*, indicating *coordination*, or *shared sense-making, decision-making*, indicating *collaboration* (see Figure 4). 

Collaboration seemed more likely to occur when professionals phoned each other (with these conversations recorded in the EHR or in an email). The first instance involving GP2 and Patient 2 was in response to a mental health crisis when the GP2 phoned [“*tagging”*] the counsellor to assess risk. GP2: *“*[I]* needed to get some counselling for her, because she needs to make some decisions quickly around this pregnancy* [to]* get some support for her mental health. So that was a clear identified need from her side, as well as a fairly obvious one from my side. And then even when she was seeing the counsellor I would be sort of tagging with the counsellor* [phoning the counsellor]* to make sure, she wasn’t deteriorating …”.*

The second instance of *collaboration,* involving the nominated professional (midwife) and Patient 2, was in response to the midwife foreseeing that she would need to collaborate with the MMH agency in relation to the care of Patient 2. Midwife:* “I also made sure that Maternal Mental Health got hold of her, because* [there shouldn’t be]* a delay in her being seen. … Yes. I chased them up. I did sort of, put a little bit more pressure* [on]*”.* This example of collaboration proved very helpful from the perspective of Patient 2, and when interviewed, she described how she was about to meet with the midwife and the mental health nurse from the MMH agency to form a joint care plan. Patient 2:* “There’s a huge overlap with my carers… Like tomorrow I’m meeting with my midwife and my mental health nurse. That immediate plan, post-natally, and to get all that in place, and to have some strategies ready”*.

### Case syntheses

The final stage of analysis combined all data sources (patient health encounter diaries, EHRs, other forms of written communication from the nominated professionals and interviews) to create expanded case syntheses involving Patient 1 and Patient 2 and the professionals.

#### Case 1

Patient 1 recorded day-to-day involvement with a very large team of traditional and non-traditional professionals, most working in separate agencies. She reported that she takes a key role in coordinating those involved in her care, and her GP agreed this was so. Patient 1 actively works on a one-to-one basis with each professional and selectively facilitates interaction between a few, stating she does not necessarily want each to be aware of the others – particularly the health professionals being aware of the CAM professionals. Although there is a large team involved, Figure 3 shows that GP1 receives information from only a small number of the professionals including the nominated professional, a physician, who also separately interacts with other traditional professionals, indicating a degree of coordination. The interaction map shows an absence of collaboration in that there is no bidirectional communication between professionals or agencies.

#### Case 2

Patient 2 recorded day-to-day involvement with a small number of professionals who worked in separate agencies. She reported a limited role with the professionals in her care, although the GP reported that Patient 2 had independently contacted her midwife. Patient 2 said she did not actively facilitate interaction between her professionals and was uncertain about whether or not this would have been useful using the qualifying phrase “*possibly at stages*”. Figure 4 shows that both GP2 and the midwife provided leadership with various interactions occurring, including bidirectional communication between the different team members, indicating collaboration between those involved in her care. Patient 2 was unaware of these extensive interactions. In two instances, collaboration between the professionals was occurring. On one occasion, Patient 2 was involved in a collaborative interaction (midwife and MMH agency) and she appreciated this form of care. 

## Discussion

The two cases studied in depth illustrate two female patients with likely similar educational levels, but who had different profiles of multimorbidity, socioeconomic circumstances, and personal capabilities. When the data from all sources were analysed, common issues emerged relating to communication between professionals in different agencies, the coordination of care, including leadership, and the level of involvement of the patient. When the various data sources were considered together, like other studies [15, 42–44], it verified the complexity of multimorbidity care from the perspective of the patients, GPs, and other professionals, and also demonstrated that the 3Cs model of consultation, coordination and collaboration is applicable to the analysis of interprofessional, interagency multimorbidity care. Coordinated, or collaborative, care is often said to occur, but in both cases in this study of naturally occurring interactions, there were many instances of consultation, but few of coordination and collaboration, demonstrating how uncommon these forms of care probably really are. Cohen *et al*. point out that each of the 3Cs is necessary to provide care and that each has its place; however, arguably, when patients with multimorbidity require care from several professionals and from different agencies, collaboration is the ideal [22]. It is significant that instances of collaboration in this study were noted and appreciated by the patients.

The CSOR methods enabled the identification of day-to-day help-seeking behaviours by the patients and the interactions between them and the general practice (particularly GPs), as well as with other professionals or agencies. Similar to other research, this study shows patients with multimorbidity can have large or small teams of professionals (some with non-traditional professionals) and include various agencies [5]. However, the current study adds to this knowledge by demonstrating the sheer number of interactions for patients, professionals, and agencies over a 12–13-week period, representing a considerable time (and likely cost) investment. It also showed that individually, and collectively, professionals/agencies were not aware of all who are involved in the patient’s care, raising questions about efficiency, gaps, or duplications of care. Research suggests that leadership is central to effective interprofessional, interagency multimorbidity care [8, 9]. In our study, even though attempts were made to provide leadership in one case, there was no formal process in either case involving a number of professionals and agencies to establish the role of a leader able to provide overall coordination, particularly when the overall team included traditional and non-traditional professionals. Although GPs are believed to be best placed to be the lynchpin of multimorbidity care, this may not be so when multiple professionals and agencies are involved, and fragmentation occurs, and in this situation they may be reluctant to assume this role [45]. Some studies, such as the one conducted by Gill *et al*. [15], have suggested that care managers or care coordination roles may be more appropriate. Similarly, a 2017 report by the Commonwealth Fund advocates making *“Care coordination a high priority”* for patients with complex needs [46]. 

The two patients presented in our study appeared to adopt very different roles with their healthcare teams; with one characterized as a ‘strong’ role and the other a seemingly ‘restricted’ role. Although not the only roles that patients adopt [47], this study shows that patients can actively facilitate, lead, gate-keep, opt-out, or become unengaged in the flow of information. Establishing how patients want to interact with professionals – particularly when several agencies are involved – is important, especially if patients actively seek to be part of the team or to be the leader. Our study showed that patients had little idea of whether or not members of their team interacted with each other, independent of them, but wanted to be informed of, or involved in, this communication. Similar to the study by Doessing and Burau [48], this also needs to be tempered with awareness that fluctuations in health status experienced by those with multimorbidity alter their ability to participate, possibly particularly so when mental health conditions or biosocial or environmental issues are part of the multimorbidity profile. Singer *et al.* believe the syndemics theory should be given attention to, in relation to multimorbidity, as it explains the unpredictability and complexity of disease interactions and why *“social, environmental or economic factors promote such interactions and worsen disease”* [4].

Mangin *et al*. [49] argue for the incorporation of decision tools that elicit patient priorities and preferences for care to be part of the clinical decision-making regarding multimorbidity, and Muth *et al*. [50] believe that this process may involve making treatment trade-offs. Our study expands on this by recommending that information on patient preferences is made available to all professionals and agencies so patients can contribute to their care, when possible, and also to allow others to enact the patient’s wishes when the patient’s illness state fluctuates and they cannot actively contribute. 

The two patients highlighted in our study used various methods to try to communicate with professionals and agencies. The most common methods were face-to-face and one-by-one interactions, which are typical approaches in New Zealand. These forms of communication are relatively expensive and time-consuming, and looking to the future, as multimorbidity increases, will likely be impractical and unsustainable, especially if large numbers of professionals are involved [6]. While some face-to-face and one-by-one interactions are essential for key interactions to be successfully undertaken, others could be replaced or augmented by other forms of communication or consultation, including e-portals, interprofessional clinics and/or videoconferencing [51]. We agree with Mercer *et al*. [52] that having a secure electronic platform for all professionals and agencies (agreed by patients) to communicate and plan care would be an advantage. Support and encouragement should be provided to patients and professionals to use these modalities [53, 54].

As with other studies [15, 43, 44], there was variability in the frequency and depth of communication between professionals; including no communication at all. There is a general agreement that multimorbidity care requires the skills of different professionals [55–57]; however, this study showed that professional-to-professional engagement, particularly when several different agencies are involved, was complex and non-uniform, with unpredictable, unidirectional, and bidirectional exchanges occurring. Non-traditional professionals are likely being excluded from communication with mainstream health professionals. Overall, this appears to be an international problem and not isolated to New Zealand [17]. Ideally, professionals within and between agencies should prioritize communication and interactions with other professionals about those with the most complex forms of multimorbidity [23]. 

### Strengths and limitations

There are strengths and limitations in this study. The case study design does not purport to produce generalizable results, rather it illustrates problems for the particular cases in their specific health system and community context [31]. The number and variety of data sources used to build each case study in this study is a clear strength [31, 58], and all proved to be necessary when triangulating the various datasets (patient, GP, other professional). Yet, analysing the data sources separately prior to integration and keeping data sources identifiable in the results provides a clear chain of evidence assuring dependability. 

Two case studies of those with multimorbidity are provided. While there is no typical profile for such a person, and social circumstance as well as personal attributes and capabilities influence how well people manage [15], ideally, in the future, this selection would be supplemented by others to elucidate a range of experiences. While this study focused on contacts with professionals, future studies should include others in the patient’s social network, including lay carers, as they are known to provide significant support [59]. 

The challenging observational health encounter diary method is likely to be even more difficult for patients with multimorbidity. It is not surprising that there was some unreliable recording of the daily diary, despite prompts and encouragement from the research team, and this is a limitation. Although GPs may have selected patients they thought would demonstrate ‘ideal interactions’, in-depth analysis showed that these interactions were variable, and the range enabled interactions to be classified according to Cohen *et al.*’s 3Cs model. The 12–13-week data collection period may not have been long enough to show or make clear any pre-existing collaborative relationships between professionals.

## Conclusion

The two patients who are described in this study contribute to our understanding of the complexity of interprofessional, interagency multimorbidity care. Each navigates a complex environment of healthcare systems, interacting with an array of healthcare professionals and others. When the patient is included in communications concerning their care, it is appreciated. Questions remain about who should lead or take responsibility for coordination of care and how this might play out in collaborative practice when health status fluctuates and varies.

Further opportunities are needed in multimorbidity research to examine patients, general practice, and other professional’s contemporaneous involvement in care, including consultation with each other, coordination of professionals and services, and cross-agency professional collaboration. There is a need to develop models of interagency care that increase the likelihood of providing coordinated or collaborative care.

## Figures and Tables

**Box 1:**
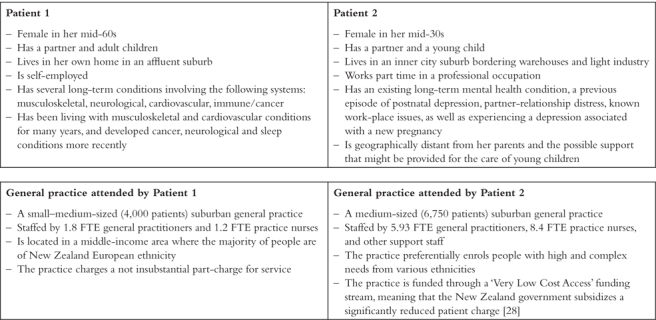
Overview of cases and general practices. FTE, full-time equivalent.

**Table 1:** Summary of patient health encounter diaries.

Patient	1	2	3	4
General practitioner	1	2	2	1
Nominated professional	Physician	Midwife	Addiction service psychologist	–
Data collection period, days	88	87	93	84
Number of daily entries	60	59	47	84

**Table 2:** Summary of contacts made between Patients 1 and 2 and professional/agencies during the 12–13-week study period, based on data obtained from the health encounter diaries.

Professional/agency	Type of contact					
	Face–face	Phone call	Letter	Email	Unknown	Total
Patient 1						55
Accident and emergency	1	1				2
Acupuncturist	1					1
After-hours clinic	1					1
Breast clinic	1			1		2
Counsellor	1					1
Dental hygienist	1					1
Dentist	3					3
Exercise physiologist	1			1		2
General practitioner 1	5	3				8
Homeopath	1					1
Masseuse	1					1
Ministry of Health			1			1
Osteopath	5					5
Pharmacist	4				1	5
Rehabilitation clinic	10					10
Sleep clinic	3		1	1		5
Sleep specialist	3		2			5
Spiritual healer	1					1
Patient 2						17
After-hours clinic	1					1
General practitioner 2	5	1				6
Maternal mental health	4	1				5
Midwife	2					2
Postnatal depression support group^*^	2					2
Radiologist	1					1

^*^Whilst not considered a health provider, the postnatal depression support group was included, as people with multimorbidity are likely to perceive it as having an equal status, especially if using a ‘trained’ facilitator.

**Table 3:** Method and frequency of contact amongst the professionals/agencies involved in the care of Patients 1 and 2, based on data obtained from electronic health records and other forms of written communication from the nominated professionals.

Sender	Main recipient	Other recipients	Method	Frequency
Patient 1				6
After-hours clinic	GP team 1^*^	–	Electronic notification	1
Radiologist	GP team 1	–	Electronic notification	1
Orthopaedic surgeon	GP team 1	Accident Compensation Corporation^¶^	Letter	1
Sleep specialist	GP team 1	Oncologist, neurologist, breast surgeon, Patient 1	Letter	2
Sleep specialist	GP team 1	Neurologist, Patient 1	Letter	1
Patient 2				22
GP team 2	Maternal mental health	–	Unknown	1
GP team 2	Multidisciplinary team meeting^#^	–	In person	1
GP team 2	Maternal mental health	–	Letter	1
Laboratory	GP team 2	–	Electronic results	1
GP team 2	Crisis assessment and treatment team	–	Letter	1
GP team 2	Crisis assessment and treatment team	–	Phone call	1
Crisis assessment and treatment team	GP team 2	–	Letter	1
GP team 2	Maternal mental health	–	Phone call	1
Radiologist	GP team 2	Laboratory, midwife	Electronic results	1
Maternal mental health	Patient 2	GP2	Letter	1
Maternal mental health	GP team 2	–	Letter	1
Unknown initiator^‡^			Phone call	1
Maternal mental health psychiatrist	Midwife	–	Email	1
Midwife	Maternal mental health psychiatrist		Email	1
Maternal mental health psychiatrist	Midwife	Maternal mental health nurse	Email	1
Maternal mental health nurse	Midwife	–	Email	2
Psychologist	GP team 2	–	Letter	1
Healthline^§^	GP team 2	–	Electronic notification	1
After-hours clinic	GP team 2	–	Letter	1
Radiologist	GP team 2	–	Electronic notification	1
GP team 2	Employer	–	Letter	1

GP, general practitioner.

*May include the GP, practice nurses and/or any other members of the practice team.

^¶^New Zealand’s 24-h, no-fault, personal accident insurance scheme.

^#^A monthly meeting in this general practice, which includes a community-based psychiatrist and several GPs and practice nurses.

^‡^Phone call between midwife and maternal mental health psychiatrist.

^§^New Zealand’s national telephone health information service.
